# Treatment and outcome of 32 patients with distant metastases of Hürthle cell thyroid carcinoma: a single-institution experience

**DOI:** 10.1186/s12885-016-2179-3

**Published:** 2016-02-26

**Authors:** Nikola Besic, Andreja Schwarzbartl-Pevec, Barbara Vidergar-Kralj, Tea Crnic, Barbara Gazic, Maja Marolt Music

**Affiliations:** Department of Surgical Oncology, Institute of Oncology, Zaloska 2, SI-1000 Ljubljana, Slovenia; Department of Nuclear Medicine, Institute of Oncology, Zaloska 2, SI-1000 Ljubljana, Slovenia; Department of Pathology, Institute of Oncology, Zaloska 2, SI-1000 Ljubljana, Slovenia; Department of Radiology, Institute of Oncology, Zaloska 2, SI-1000 Ljubljana, Slovenia

**Keywords:** Thyroid carcinoma, Treatment, Radioiodine, Hürthle cell thyroid carcinoma, Surgery, Radiotherapy, Outcome, Pathology

## Abstract

**Background:**

It is generally believed that patients with Hürthle cell thyroid carcinoma (HCTC) have a poor prognosis. Furthermore, distant metastases represent the most frequent cause of thyroid cancer-related death of patients with HCTC. The aim of this study was to report the treatment and outcomes of patients with distant metastases.

**Methods:**

Altogether 108 patients were treated for HCTC from 1972 to 2011 in our tertiary center and 32 patients (19 females, 13 males; median age 64.5 years) had either initially proven metastatic disease (*N* = 12) or distant progression of HCTC after initial treatment (*N* = 20). Patients with metastases were followed for 1–226 (median 77) months. Data were collected on the patients’ gender and age, extent of their disease, morphologic characteristics, therapy, outcome, and survival rate. Statistical correlation between possible prognostic factors and cause-specific survival from time of detection of metastases was analyzed by univariate analysis and log-rank test.

**Results:**

The most common were lung metastases, followed by bone, mediastinum, kidney, and liver in 24, 8, 2, 1, and 1 case, respectively. Total thyroidectomy, lobectomy, subtotal thyroidectomy and neck dissection were performed in 19, 10, 3, and 7 patients, respectively. Radioiodine (RAI) ablation of thyroid remnant was performed in 30 patients, while 20 of them had RAI therapy (median 4 times). RAI uptake in metastases was present in 16 patients and ranged from 0.05 % to 12 %. Chemotherapy was used in 13 patients and external beam radiotherapy in 19 patients. Locoregional control of disease was achieved in 19/21 (90 %) cases who succumbed due to HCTC. Estimated 10-year disease-specific survival for all patients was 60 %. 10-year disease-specific survival for patients with pulmonary metastases and other sites metastases was 60 % and 62 %, respectively. 10-year disease-specific survival for patients with single organ and multiple organ metastases was 52 %, and 100 %, respectively. Estimated median disease-specific survival after the diagnosis of metastatic disease for all patients was 77 months. The median disease-specific survival after the diagnosis of metastatic disease for patients with pulmonary metastases and other sites metastases was 72 and 138 months, respectively.

**Conclusions:**

Ten-year disease-specific survival for all patients with metastatic Hürthle cell thyroid carcinoma, patients with pulmonary metastases and bone metastases was 60 %, 60 % and 68 %, respectively.

## Background

Hürthle cell thyroid carcinoma (HCTC) is regarded to be an oxyphilic variant of follicular thyroid cancer (FTC) according to the World Health Organization classification [1]. However, genomic studies revealed that HCTC is a unique thyroid malignancy distinct from papillary and follicular thyroid cancer [2]. HCTC is a rare type of thyroid carcinoma [3] which comprises about 3 % of all thyroid malignancies [4].

Only about 400 patients with HCTC were reported from 1935 to 2004 [3]. There are several reports of single-institution studies of patients with HCTC [5–23], but only two recently published single-institution series included more than 100 patients [22, 23]. Older studies reported poor survival of patients with HCTC [5, 6, 8–10]. But in population-based studies from the United States, Nagar et al. [24] and Bhattacharyya [2] reported that the survival of patients with HCTC has improved dramatically over time and that survival rates of HCTC and FTC are currently the same. Unfortunately, the treatment of patients was not reported and causes of prolonged survival were not explained.

In 2003, we reported that the uptake of radioiodine (RAI) was confirmed in 11 of 16 patients with metastatic HCTC [15]. The purpose of the present study was to report the uptake of RAI in a larger number of patients with metastatic HCTC. Another of this study’s aims was to describe the treatment and outcome of 32 patients with metastatic HCTC with a long follow-up period.

## Methods

Altogether 108 patients were treated for HCTC at our tertiary center from 1972 to 2011, and 32 patients (19 females, 13 males; median age 64.5 years) had either an initially proven metastatic disease (*N* = 12; 9 females, 3 males; median age 66.5 years) or distant progression of HCTC after initial treatment (*N* = 20; 10 females, 10 males; median age 64 years).

The Medical Ethics Committee of the Republic Slovenia and The Protocol Review Board and Ethics Committee of the Institute of Oncology approved the study, which was conducted in accordance with the ethical standards laid down in an appropriate version of the 1964 Declaration of Helsinki. The need for consent was waived by the Institutional Review Board and Ethics Committee of the Institute of Oncology Ljubljana. For retrospective studies a written consent is deemed unnecessary according to national regulations.

As already reported in one of our previous studies [23], all histological slides of our patients with HCTC were examined by the pathologist (B.G.) experienced in thyroid pathomorphology. HCTC was diagnosed on the histological criteria defined by LiVolsi and Rosai [25, 26]. An oncocyte was characterized by the presence of acidophilic, granular cytoplasm and hyperchromatic or vesicular nuclei with large nucleolus. Only lesions demonstrating more than 75 % of follicular cells with oncocytic characteristics were included in the study group. All patients with Hürthle cell neoplasms with cells containing typical nuclear features of papillary carcinoma were excluded from our present study and were the subject of one of our previous studies [27]. The diagnosis of malignancy for present study was based on histologic evidence of transcapsular and/or vascular invasion, extrathyroidal local tissue invasion by the primary tumor [25, 26], or presence of nodal or distant metastasis.

A chart review was performed, and data on patients’ age, gender, disease history, extent of disease, histomorphological characteristics, mode of therapy, outcome, and survival were collected. Clinical characteristics, treatment of patients and their outcome are presented in Table 1, Table 2 and Table 3.

**Table 1:** Patients’ and tumor characteristics and univariate statistical analysis of disease-specific survival

Factor	Subgroup	Initially metastatic (*N* = 12)	Distant progression (*N* = 20)	*p*-value
Gender	Female	9	10	0.16
	Male	3	10	
Age at presentation (years)	64 or less	6	5	0.15
	65 or more	6	15	
Tumor diameter (cm)	0-4	3	4	0.74
	4.01 and more	9	16	
pT tumor stage	pT0 or pT2	2	3	0.96
	pT3	6	11	
	pT4	4	6	
N stage	N0	10	16	0.48
	N1 (N1a + N1b)	2	4	
M stage	M0	12	0	-
	M1	0	20	
Thyroid surgical procedure	Total or near-total thyroidectomy	6	13	0.40
	Lobectomy or less	6	7	
Neck dissection	No	9	18	0.26
	Yes	3	2	
Residual tumor after surgery	R0 – without residual tumor	3	12	0.066
	R1 – microscopic residual tumor	8	5	
	R2– macroscopic residual tumor	1	3	
Radioiodine ablation after surgery	No	1	1	0.71
	Yes	11	19	
Therapy with radioiodine	No	5	8	0.93
	Yes	7	12	
External beam irradiation - neck	No	6	14	0.40
	Yes	6	6	
External beam irradiation – any site	No	2	11	0.03
	Yes	10	9	
Chemotherapy	No	4	16	0.008
	Yes	8	4	
Outcome	Alive without disease	0	0	-
	Alive with disease	2	9	
	Dead of disease	9	11	
	Dead of other causes	0	0	
	Lost to follow-up	1	0

**Table 2:** Clinical characteristics of 32 patients and their outcome

Patient number	Age range at presentation	Age range at metastases	Tumor diameter (cm)	pT-stage	pN-stage	M-stage	Site of metastases	Tg at diagnosis of metastases	Survival	Disease-free interval	Survival from diagnosis of distant metastases	Cause of death
1	85–89	85–89	7,5	3	0	1	L	7414	77	0	77	DDM
2	75–79	75–79	5	4b	0	1	L	4450	53	0	53	DOC
3	70–74	70–74	8	3	0	1	L	265	141	0	141	DDM
4	70–74	70–74	6	4a	0	1	B	183	13	0	13	DDM
5	65–69	65–69	8,5	3	0	1	L	16650	185	0	185	Alive
6	65–69	65–69	8	3	0	1	BL	4010	148	0	148	DDM
7	60–64	60–64	8	0	0	1	B	No data	153	0	153	DDM
8	60–64	60–64	4,5	3	1b	1	L	62600	131	0	131	DDM
9	60–64	60–64	2	2	0	1	B	69	1	0	1	LFU
10	60–64	60–64	6,5	3	0	1	L	1300	72	0	72	DDM
11	55–59	55–59	5	4b	1b	1	L	190	201	0	201	DDM
12	45–49	45–49	9	4b	0	1	L	5800	52	0	52	DDM
13	85–89	85–89	12	3	0	0	Mediastinum	277	52	38	14	DDM
14	75–79	75–79	3	2	0	0	B	47	126	34	92	Alive
15	75–79	85–89	8	3	0	0	L	696	144	84	60	DDM
16	70–74	70–74	2	4a	1a	0	Kidney	1432	318	180	138	DDM
17	70–74	75–79	8	3	0	0	L	1300	63	36	2	DDLTP
18	70–74	80–84	6,8	3	0	0	L	1000	132	96	36	Alive
19	70–74	70–74	5,5	4a	0	0	L	No data	26	3	23	DDM
20	65–69	65–69	3,6	3	0	0	L	0.1	46	43	3	Alive
21	65–69	70–74	7	4a	0	0	L	No data	63	53	10	DDM
22	65–69	65–69	9	3	0	0	BL	1100	145	52	93	DDM
23	60–64	65–69	18	4b	1b	0	L	416	100	20	53	DDM
24	60–64	70–74	3	2	0	0	L, liver	501	121	84	36	DDM
25	60–64	65–69	4,5	3	0	0	L	30773	97	49	48	Alive
26	60–64	70–74	7	3	0	0	L	196	138	113	27	Alive
27	60–64	60–64	6,5	3	1a	0	B	763	43	16	27	Alive
28	60–64	60–64	8	4a	0	0	Mediastinum	1800	145	44	101	DDLTP
29	50–54	55–59	13	4b	0	0	L	800	75	26	42	DDM
30	50–54	75–79	6	3	0	0	L	163	337	31	65	DDM
31	50–54	50–54	3	2	1b	0	L	Tg-antibodies	33	9	24	Alive
32	35–39	40–44	4	3	0	0	BL	108	286	60	226	Alive

Legend: *B* bone, *L* lungs, *DDM* dead of distant metastases, *DOC* dead of other causes, *LFU* lost to follow-up, *DDLTP* dead of distant and locoregional tumor progression

**Table 3:** Treatment of patients

Patient number	Initial surgery	Residual tumor after surgery	RAI ablation of thyroid remnant	RAI uptake	RAI applications: ablation and therapy (Number)	RAI cumulative dose (mCi)	Neoadjuvant ChT	ChT	Preoperative EBRT (Dose in Gy)	EBRT - neck and mediastinum (Dose in Gy)	EBRT of metastases	Surgery for metastases or recurrence
1	TT	R0	No	-	0	-	No	No	No	No	No	No
2	LO	R1	Yes	No	1	129	No	No	No	Yes (50)	No	No
3	LO	R1	Yes	Yes	7	1066	No	No	No	Yes (50)	No	No
4	LO	R2	Yes	No	1	94	Yes	Yes	Yes (36)	Yes (36 + 15)	Ribs, thorax	No
5	TT	R1	Yes	Yes	4	500	Yes	Yes	No	Yes (56)	Lumbal vertebra, left hip and left pelvis	No
6	LO	R1	Yes	Yes	8	1201	Yes	Yes	No	No	Thoracic vertebra - rwice	No
7	PT	R0	Yes	No	2	250	No	Yes	No	No	Frontal part of skull, left orbita	Resection of right clavicle
8	TT + RND	R1	Yes	No	1	100	Yes	Yes	No	No	Lungs twice	No
9	TT	R0	Yes	No	1	94	No	No	No	No	No	No
10	TT + RND	R1	Yes	Yes	8	1355	Yes	Yes	No	Yes (50)	No	No
11	LO + RND	R1	Yes	Yes	8	1156	Yes	Yes	No	Yes (45)	Left hip twice, thorax, right hip, brain, right clavicle	No
12	TT	R1	Yes	Yes	9	1278	Yes	Yes	No	No	Thoracic vertebra, pelvis, cervical vertebra	Laminectomy
13	TT	R1	No	-	0	-	No	No	No	No	No	No
14	TT	R0	Yes	Yes	5	779	No	No	No	No	No	No
15	TT	R0	Yes	No	1	92	No	No	No	No	No	Left hip replacemnt
16	LO	R0	Yes	Yes	6	1090	No	No	No	No	No	Right nephrectomy
17	TT	R0	Yes	No	1	106	No	No	No	No	No	No
18	TT	R0	Yes	No	1	261	No	No	No	No	No	No
19	LO	R0	Yes	Yes	2	350	No	No	No	No	No	No
20	TT	R2	Yes	No	1	100	No	No	No	Yes (50)	No	Tracheal shaving of recurrence
21	PT	R2	Yes	Yes	2	230	No	No	No	No	No	No
22	TT	R0	Yes	Yes	2	300	No	No	No	No	Base of skull, lungs, right neck	No
23	TT + RND	R1	Yes	No	1	100	Yes	Yes	No	Yes (49)	Clavicle	No
24	PT	R1	Yes	Yes	9	1296	No	No	No	Yes (50)	No	No
25	TT	R0	Yes	No	2	244	No	No	No	No	No	No
26	TT	R0	Yes	Yes	3	382	No	No	No	No	No	No
27	TT + RND	R1	Yes	No	1	95	No	Yes	No	Yes (50)	Sternum, clavicle, right tibia	No
28	LO	R1	Yes	Yes	11	1523	No	Yes	No	Yes (50)	No	No
29	LO	R2	Yes	No	2	263	Yes	Yes	No	Yes (51)	Mediastinum, lungs	Tracheostomy, mRND
30	LO	R0	Yes	Yes	2	230	No	No	No	No	Mediastinum, lungs	Resection of lung metastasis and twice brain matastasis
31	TT	R0	Yes	Yes	3	393	No	No	No	No	No	Parotidectomy, mRND
32	TT	R0	Yes	No	1	95	No	Yes	No	No	Pelvis, left elbow, lumbar vertebra	6 x lung metastasis, 5 x bone metastasis, mediastinal lymph nodes, liver metastasis, axillary lymphadenectomy

Legend: *TT* total or near total thyroidectomy, *LO* lobectomy, *PT* partial thyroidectomy, *RND* modified radical neck dissection, *RAI* radioiodine, *ChT* chemotherapy, *EBRT* external beam radiotherapy

The tumor stage, presence of regional and/or distant metastases, as well as residual tumor after surgery were assessed by the TNM clinical classification according to the UICC criteria from 2009 [28]. The initial diagnostic work-up in all patients with HCTC included ultrasound (US) of the neck region, determination of the serum thyroglobulin (Tg) concentration and chest X-ray. The criteria for disease-free survival were Tg levels of less than 1 ng/mL, negative whole-body RAI scans, and the exclusion of cervical lymph node metastases by US. Additional diagnostic work-up (X-ray, US, computed tomography, magnetic resonance imaging, bone scintigraphy, scintigraphy with MIBI and/or PET-CT) was conducted whenever the medical history, physical examination, laboratory findings, and/or radioiodine (RAI) uptake indicated recurrence and/or distant metastases.

All patients with HCTC had a follow-up exam at our Institute at least once per year, while for all patients with distant metastases follow-ups were conducted at least twice per year. This consisted of obtaining medical history, a physical examination, and determining serum Tg concentration. Imaging was also conducted whenever Tg concentration increased or clinical symptoms suggested that the disease had progressed.

### Treatment

During 30 year period, surgical treatment of primary tumor, locoregional recurrences, and distant metastases was not uniform; neither was the proportion of patients treated by RAI ablation of thyroid remnant tissue, external beam radiotherapy (EBRT), chemotherapy and RAI therapy [23]. Surgery is the mainstay of the treatment of HCTC. Among surgically treated patients, 84 % had primary surgery at the Institute of Oncology and 16 % elsewhere, while all other specific therapies (surgery, RAI, EBRT and/or chemotherapy) as well as follow-up were conducted for all patients at the Institute of Oncology. After surgery, all patients received therapy with L-thyroxin for thyrotropin (TSH) suppression.

The data on the type of surgery for primary tumors and treatment of distant metastasis are listed in Table 3. Total or near-total thyroidectomy is considered a proper surgical procedure for HCTC. Generally, after an inadequate surgical procedure a completion thyroidectomy is performed. However, in our study group this was performed in only one of 13 patients with inadequate surgery. It was not performed if the patient had an injury of recurrent nerve (*N* = 2), or if the patient was very old or with severe comorbidities (*N* = 2), if the patient refused another surgical procedure (*N* = 2), and preferred treatment with RAI (*N* = 6).

Initial treatment in nine patients with a locally advanced tumor was neoadjuvant chemotherapy, while one patient received concomitant EBRT with a tumor dose of 36 Gy. Tumor size decreased in all of these patients, and in 2 of 9 patients the largest tumor diameter decreased by more than 30 %. Neoadjuvant chemotherapy consisted of long infusion of low dose vinblastine (2 mg in 12-h) in four patients and of vinblastine and doxorubicin (20 mg in 2-h infusion) in two patients. Neoadjuvant chemotherapy consisted of vinblastine, doxorubicin and mitoxantrone in one patient, while in two patients more than three cytostatic drugs were used (vinblastine, doxorubicin, docetaxel and cisplatin in one patient; vinblastine, cisplatin, doxorubicin, bleomycin, cyclophosphamide, mitoxantrone in another one).

Metastases in regional lymph nodes were treated surgically by functional radical neck dissection in five as part of primary treatment. Surgical therapy of metastatic disease and/or locoregional recurrence was conducted on nine patients (Table 3). It included neck dissection, resection of lung metastasis, resection of bone metastasis, resection of brain metastasis, resection of liver metastasis, nephrectomy, laminectomy, hip replacement, superficial parotidectomy, tracheal shaving, and tracheostomy.

RAI was used for the ablation of thyroid remnant tissue in 30 (94 %) patients. All 13 patients with non-adequate surgery had the ablation of thyroid remnant tissue. The ablation dose was 3.4–4.8 GBq (92–129 mCi) of RAI. RAI was used also for treating of distant metastases and inoperable locoregional recurrences with empiric dose of 3.7–7.4 GBq (100–200 mCi). Patients with distant metastases underwent total thyroidectomy prior to treatment with RAI in order to increase the uptake of iodine in metastases and to speed up the treatment whenever possible. Before 2002, serum concentration of TSH of >30 mU/L was achieved by a 4–6-week withdrawal of L-thyroxin suppression therapy. Since 2002, recombinant human TSH (rhTSH)-aided RAI therapy [29] has been used in altogether eight patients who were elderly with concomitant diseases, had a history of severe hypothyroid or compressive symptoms and/or evidence of tumor progression during thyroid hormone withdrawal.

Altogether 13 patients were treated with chemotherapy. Kinase inhibitors were not used in patients included in the present report.

EBRT was done in a total of 19 patients, of whom twelve received EBRT to the neck and superior mediastinum (3 after R2 resection and 9 after R1 resection).

### Survival

Cause-specific survival was defined as the period from primary treatment (surgery, chemotherapy or EBRT) to death or the last follow-up.

Disease-free survival was defined as the period from primary treatment (surgery, chemotherapy or EBRT) to the radiologic or morphologic diagnosis of recurrence or the last follow-up. The median duration of follow-up was 8.3 years (range 0.1–28.1 years).

### Statistical analysis

The Student *t*-test or the Mann–Whitney *U*-test was used according to data distribution. The association between categorical variables was tested by the chi-square test or Fisher’s exact test, as appropriate. All comparisons were two-sided and a *p*-value <0.05 was considered statistically significant. The statistical correlation between possible prognostic factors and cause-specific survival was analyzed by chi-square test and log rank analysis. The survival curves were calculated according to the Kaplan-Meier method. A multivariate statistical analysis was not performed because of the small number of patients. The statistical package PASW 18 (SPSS Inc., Chicago, IL, USA) was used for the analysis.

## Results

The median age of patients with initially proven metastatic disease (*N* = 12) and distant progression of HCTC after initial treatment (*N* = 20) was 66.5 and 64 years, respectively. The median age of both groups of patients was not statistically different. A female-to-male ratio in initially proven metastatic disease and distant progression after initial treatment was 3:1 and 1:1, respectively. Gender distribution in both groups of patients was not statistically different (*p* = 0.27).

The tumor diameter ranged from 2 to 18 cm (median 6.65 cm, mean 6.75 cm). Median tumor size in initially metastatic disease and distant progression was 7 cm and 6.65 cm, respectively (*p* = 0.75). Extrathyroid tumor growth was present in patients with and without initially proven metastatic disease and 75 % and 40 %; respectively (*p* = 0.27).

Single organ and multiple organ metastases at the time of diagnosis of dissemination were diagnosed in 28 and 4 cases, respectively. The most common were lung metastases, followed by bone, mediastinum, kidney, and liver in 24, 8, 2, 1, and 1 case, respectively. Lung metastases were present in patients with initially proven metastatic disease and in patients with metastases after a disease-free interval in 75 % (*p* = 1.0). Bone metastases were present in patients with initially proven metastatic disease and in patients with metastases after a disease-free interval in 33 % and 20 %; respectively (*p* = 0.43).

Median Tg concentration at the diagnosis of metastases was 1050 ng/mL (range 47–62600 ng/mL).

### Treatment

The data on the type of treatment of patients are summarized in Table 1. All patients had surgical treatment on their primary tumor. Total (or-near total) thyroidectomy, lobectomy, subtotal thyroidectomy, and neck dissection were performed in 19, 10, 3, and 7 patients, respectively. RAI ablation of thyroid remnant was performed in 30 patients, while RAI therapy was performed in 20 of them. They received from 1 to 10 RAI therapies (median 4) and cumulative RAI dose ranged from 6.21 to 41.16 GBq (230 to 1523 mCi) with a median cumulative dose of 24–96 GBq (922 mCi). RAI uptake in metastases was present in 16 patients and ranged from 0.05 % to 12 % (median 0.5 %). In 9 of patients the uptake of RAI was 0.5 % or larger. Chemotherapy and EBRT was used in 13 and 18 patients, respectively.

The treatment of metastases was heterogeneous. Two patients had surgery because of lung metastases. In the first one a lung metastasis was excised because it was solitary and occurred after a long disease-free interval. The other patient had six surgical procedures because of small multiple metastases which were resected at other hospital because the patient preference and persistence. The same patient had also numerous surgical procedures of bone metastases. On the other hand, majority of bone metastases in our patients lesions were efficiently treated by EBRT. In only two patients a pathologic bone fracture necessitated surgical approach: a right hip replacement because of fracture of femur and two surgical procedures of the right ulna. Two patients had liver metastases: one of them was treated with RAI eight times and there was an uptake in liver metastases. The other patient had pulmonary and liver metastases and RAI uptake was observed in liver three times. Because of progression of liver metastases and T3-hypethyreosis she was treated also with chemotherapy (vinblastine and doxorubicin, vinblastine and cisplatin) with only partial response.

### Disease-free survival and disease-specific survival

Following initial treatment, distant metastases were proven in 20 patients after follow-up of 3 to 180 months (median 44 month). Distant dissemination was diagnosed as only distant, both locoregional and distant, and after surgical treatment of locoregonal recurrence in 11, 3, and 6 cases, respectively.

Nine patients are alive with a median follow-up of 33–286 months. One patient was lost from follow-up. One patient died of causes unrelated to primary disease, while 21 patients died of thyroid carcinoma. Of the latter, 19 patients died of distant metastases, while two patients died of distant and locoregional progression of the disease. Locoregional control of the disease was achieved in 19/21 (90 %) patients who succumbed due to HCTC.

Table 4 shows details about median length of disease-specific survival from the diagnosis of metastatic disease, 5-year disease-specific survival and 10-year disease-specific survival for all patients, those with pulmonary, bone, single organ, multiple organ metastases, initially metastatic, and metastatic after disease-free interval.

**Table 4:** Median estimated disease-specific survival, 5-year disease-specific survival and 10-year disease-specific survival from inital treatment and from the time of diagnosis of metastatic disease

	From the initial treatment			From the diagnosis of distant metastases		
	Estimated median length of survival (months)	Estimated 5-year disease-specific survival (%)	Estimated 10-year disease-specific survival (%)	Estimated median length of survival (months)	Estimated 5-year disease-specific survival (%)	Estimated 10-year disease-specific survival (%)
All patients	145	81	60	77	54	40
Pulmonary metastases all patients	141	81	60	72	52	31
≤3 pulmonary metastases	100	67	44	32	27	-
>3 pulmonary metastases	141	77	55	76	70	35
Without pulmonary metastases	153	74	62	138	55	37
Bone metastases	148	70	68	148	70	50
Single organ metastases	131	77	52	72	58	37
Multiple organ metastases	145	100	100	93	70	38
Initially metastatic	141	76	52	141	76	52
Metastatic after disease-free interval	145	81	60	65	44	19

Patients treated with neoadjuvant chemotherapy had a more advanced stage of disease than those who were not treated by neoadjuvant chemotherapy (Table 2 and Table 3). Estimated median disease-specific survival from the beginning of treatment in patients with and without neoadjuvant chemotherapy was 100 and 141 months (log-rank *p* = 0.256), respectively. 5-year disease-specific survival for patients with and without neoadjuvant chemotherapy was 78 % and 90 %, respectively. Median disease-specific survival from the diagnosis of metastatic disease in patients with and without neoadjuvant chemotherapy was 93 and 72 months, respectively (log-rank *p* = 0.89).

Median disease-specific survival from the initial treatment in patients with and without total thyroidectomy was 145 months in both groups of patients. Median disease-specific survival from the time of diagnosis of distant metastases in patients with and without total thyroidectomy was 93 months and 65 months (*p* = 0.32), respectively.

Median disease-specific survival from the initial treatment of patients with and without RAI therapy was 145 months and 100 months, respectively. Median disease-specific survival from the diagnosis of distant metastases in patients with and without RAI therapy was 93 months and 77 months, respectively. Patients treated only with surgery and RAI without EBRT and/or chemotherapy had median disease-specific survival 49 months from the initial therapy and 27 months from the diagnosis of distant dissemination.

## Discussion

HCTC is a rare disease, so there are only limited data about treatment of patients with metastatic and/or locally advanced HCTC [3, 6, 7, 9, 11, 14–17, 20, 23, 29, 30] because the majority of series with HCTC included only a very limited number of patients. To our knowledge this study represents the largest group of patients with distant metastases of HCTC and long follow-up in all the literature. Large primary tumors in our patients probably accounted for the high frequency of metastatic disease in our population [23]. Since the data were gathered over 39 years, many of the early cases were prior to the current epidemic of diagnosis of small and limited tumors. But a limitation of our study is that treatment was not uniform. Another limitation of our study is that only one of 13 patients with inadequate surgery underwent completion surgery, including six patients who “preferred treatment with RAI”.

Total thyroidectomy and RAI ablation of thyroid remnant tissue have an impact on earlier detection of recurrence and disease-free survival and possibly also on cause-specific survival of patients with HCTC [23]. Patients with HCTC from different centers were treated with various approaches. Total or near-total thyroidectomy was conducted on patients reported by Kushchayeva et al. [1], Har-El at al. [7], Chindris et al. [22], Petric et al. [23], Khafif et al. [11], Mills et al. [20], Lopez-Penabad et al. [16], and Stojadinovic et al. [14] in 90 %, 82 %, 81 %, 71 %, 64 %, 52 %, 47 %, and 30 % of cases, respectively. Additionally, RAI ablation of thyroid remnant tissue was conducted in those reported by Chindris et al. [22], Petric et al. [23], Kushchayeva et al. [1], Mills et al. [20], Har-El at al. [7], and Khafif et al. [11] in 87 %, 80 %, 64 %, 44 %, 18 %, and 9 %, respectively. EBRT to the neck region was conducted in 28 %, 23 %, 21 %, 18 %, and 2 % of patients reported by Lopez-Penabad et al. [16], Mills et al. [20], Petric et al. [23], Kushchayeva et al. [1], and Chindris et al. [22], respectively. The 10-year disease-specific survival was 88 % [23] and only 49 % in a study reported by Petric et al. [23] and Kushcayeva et al. [1], respectively. However, it should be stressed that the survival rates represent the result of both selection bias, i.e. more advanced disease in larger centers, and the effectiveness of different treatment modalities used in patients.

Our patients with metastatic HCTC had suppressed TSH with L-thyroxin. Other treatment options for metastatic HCTC are RAI, EBRT, surgery, chemotherapy, and/or targeted therapy. There is a paradigm that metastases of HCTC do not accumulate RAI. However, in 1994 Caplan et al. [9] reported normalization of elevated Tg concentration in two cases of HCTC after treatment with RAI [9]. Furthermore, in 2003, our group reported the uptake of RAI (range 0.1–12 %) in 11 of 16 (69 %) patients with distant metastases of HCTC from our tertiary comprehensive cancer center [15]. The updated data is presented in the current publication. RAI uptake was present in metastases in 16 of 30 patients (53 %) who had distant metastases. Uptake of RAI was 0.5 % or higher in 9 of 30 patients (30 %). Similarly, Lopez-Penabad et al. reported that 38 % of the patients with known metastases of HCTC showed RAI uptake in the period between 1944 and 1995 at the MD Anderson Cancer Center [16]. However, former data from Mayo Clinic are in disagreement with results from MD Anderson Cancer Center and our institute [22]. At Mayo, RAI scintigraphy (either TSH-stimulated or in hypothyroid state) was performed in 40 patients with metastases and RAI uptake was seen in only 9 cases (22.5 %) [22]. A possible reason for discrepant results may be the widespread use of CT and PET-CT with iodine radio-contrast media in the diagnostic work-up of patients with suspected dissemination of HCTC in the States.

RhTSH-aided RAI may be effective in RAI avid metastatic HCTC [29]. Numerous RAI-avid lesions were detected after rhTSH aided RAI therapy in five of six of our patients with HCTC [29]. Furthermore, 2 of 6 patients had partial response and one of them was long lasting [29]. Our view is therefore that RAI therapy may be effective treatment option in patients with HCTC which should not be neglected in patients with RAI avid metastases. Treatment with RAI has low toxicity and price in comparison to the treatment with tyrosine kinase inhibitors. We believe that they may be treatment of choice when metastases are non-RAI avid. But studies should be performed to assess the role of tyrosine kinase inhibitors in Hurthle cell carcinoma that is not radio-iodine avid.

Also EBRT may be an effective treatment option in metastatic HCTC. In 1986, Har-El et al. reported that after incomplete resection and EBRT of inoperable neck mass a patient survived 14 years [7]. Foote et al. from the Mayo Clinic stated that HCTC is a radiosensitive tumor [17]. Our data support their observation that postoperative EBRT prevents the recurrence of advanced resected tumors [17]. EBRT was conducted in a total of 19 patients, of whom twelve received EBRT to the neck and superior mediastinum and long-lasting locoregional control of disease was achieved in all three patients after macroscopic residual tumor resection. Furthermore, our experience confirms observation from Mayo Clinic that EBRT provide palliative relief from symptomatic metastases [17]. The effect of EBRT of bone metastases in our patients lasted from 13 to 165 months (median 93 months).

Surgical procedure is another useful treatment option for distant metastasis in selected cases. In our patients, surgical therapy of metastatic disease and/or local recurrence included functional neck dissection, resection of lung, bone, brain and liver metastasis, nephrectomy, laminectomy, hip replacement, superficial parotidectomy, tracheal shaving, and tracheostomy. Khafif et al. reported that one patient who had a resection of a lung metastasis was alive and free of disease 18 years after his initial surgery [11].

Our previously published data showed that chemotherapy may be effective in primary HCTC [30]. Chemotherapy in neoadjuvant setting decreased size of primary HCTC in 43 % of cases [30]. Lopez-Penabad et al. reported that systemic chemotherapy was administered to 27 % of patients, mainly those with unresectable or progressive metastatic disease [16]. Thirteen percent of patients received chemotherapy initially as a radiosensitizing agent, and the remaining 87 % of patients received a full course of treatment [16]. The agents that were used most commonly were doxorubicin alone in 43 % of patients, doxorubicin plus cisplatin in 38 % of patients, cisplatin alone in 10 % of patients, and an unspecified agent in the reviewed records of 9 % of patients [16].

There is a bias about performing a total thyroidectomy and duration of survival in our group of patients. Larger proportion of patients without a total or completion thyroidectomy had more advanced disease, so they were treated with EBRT, chemotherapy and radioiodine more frequently than patients after a total thyroidectomy. Interestingly, estimated median disease-specific survival from the initial treatment in patients with and without total thyroidectomy was 145 months in both groups of patients. Furthermore, there was no statistically significant difference in median disease-specific survival of both groups of patients from the time of diagnosis of distant metastases. This suggests possible impact of more aggressive combined treatment on survival of patients.

Pittas et al. reported that nine patients with bone metastases of HCTC have longer survival in comparison to 74 patients with bone metastases of follicular, papillary, or undifferentiated thyroid carcinoma [31]. Our results confirm their observation that patients with metastatic HCTC have a long survival. Median disease-specific survival from the diagnosis of metastatic disease for our 8 patients with bone metastases was 148 months. Five-year and ten-year disease-specific survival was 70 % and 50 %, respectively. It is interesting that our four patients with multiple organ metastases had 100 % 5-year survival from the first treatment and 75 % from the time of diagnosis of distant dissemination. It should be stressed that, the number of patients with multiple organ metastases in our series is very small. Survival of larger series of patients with multiple metastases should be analyzed in order to find out if these patients have more favorable prognosis in comparison to solitary organ metastases. Most likely these four patients had more slowly progressing tumor in comparison to other 28 patients. The other possible explanation is that their tumor was very responsive to treatment: all four had RAI ablation of thyroid remnant, three had therapy with chemotherapy and RAI and all four had EBRT.

Longer survival of patients with HCTC in comparison to other types of differentiated thyroid carcinoma is probably due to more indolent course of HCTC [31]. However, also HCTC is a progressive disease, so treatment of recurrent and/or metastatic HCTC is necessary in all those patients who are not extremely old or with severe concomitant diseases.

## Conclusions

Ten-year disease-specific survival for patients with metastatic HCTC was 60 %. Patients with bone metastases live longer than patients with lung metastases.

### Consent

Written informed consent was obtained from the patient for the publication of this report and any accompanying images.

### Availability of data and materials

There are no additional data or materials to be shared.

## Abbreviations

B: bone ChT, chemotherapy
DDLTP: dead of distant and locoregional tumor progression
DDM: dead of distant metastases
DOC: dead of other causes
EBRT: external beam radiotherapy
FTC: follicular thyroid cancer
GBq: gigabecquerel
HCTC: Hürthle cell thyroid carcinoma
LFU: lost to follow-up
mCi: millicurie
L: lungs
LO: lobectomy
MIBI: methoxyisobutylisonitrile
PET-CT: positron emission tomography and computerized tomography
PT: partial thyroidectomy
RAI: radioiodine
rhTSH: recombinant human thyrotropin
RND: modified radical neck dissection
R1: microscopic residual tumor
R2: macroscopic residual tumor
Tg: thyroglobulin
TNM: tumor, lymph nodes, distant metastases
TSH: thyrotropin
TT: total or near total thyroidectomy
UICC: Union for International Cancer Control
US: ultrasound
